# Nickel dynamics and immobilization in soil-bauxite residue systems: insights from sequential extraction and FTIR analysis

**DOI:** 10.1007/s11356-025-36701-z

**Published:** 2025-07-05

**Authors:** Ioannis Zafeiriou, Dafni Ioannou, Panagiotis Angelopoulos, Evgenia Georgiou, Dionisios Gasparatos, Ioannis Massas

**Affiliations:** 1 Laboratory of Soil Science and Agricultural Chemistry, Department of Natural Resources Management & Agricultural Engineering, School of Environment & Agricultural Engineering, Agricultural University of Athens, 11855 Athens, Greece; 2 Laboratory of Metallurgy, School of Mining and Metallurgical Engineering, National Technical University of Athens, Zografou Campus, 9 Iroon Polytechniou Str., Zografou , 15780 Athens, Greece

**Keywords:** Bauxite residue, Nickel, Adsorption, Sequential extraction, FTIR, Soil

## Abstract

Bauxite residue (BR) or “red mud” is an industrial by-product that its cumulative total reached 4 billion tonnes by 2022. Developing alternative management strategies is a matter of crucial significance on a worldwide scale. According to many references, BR has been proven an efficient barrier of metal mobility in soil environments. In this study, the results of BR addition 20% w/w in six soils with different properties on Ni mobility are presented. The results indicate that BR could be used in some cases as a Ni barrier. More specifically, BR increased pH values of all soils by approximately 2 units, leading to pH values > 9 for alkaline soils while for the acid soil, pH < 8.5 was recorded. Ni sorption on soils and soil-BR mixtures was tested for initial concentrations of 1 to 90 mg Ni L^−1^. Soil properties governed Ni sorption on soils that was much higher for alkaline or neutral soils. On the contrary, BR impressively increased Ni sorption on soil-BR mixtures, up to 24.5, 71.8, and 204%, for alkaline, neutral, and acid soils, respectively, clearly emphasizing the dominant role of BR on Ni mobility. Immediately after Ni sorption, soil and soil-BR-mixture samples were subjected to sequential extraction following the Tessier protocol for Ni fractionation. In alkaline soils, almost 50% of sorbed Ni was found in the residual fraction at the lowest initial Ni concentration that was gradually reduced as initial Ni concentration increased leading to an increase of the exchangeable fraction up to 40%. In acidic soil, the exchangeable fraction included over 50% of sorbed Ni in the widest range of tested initial Ni concentration. The addition of BR in soils reversed the picture since regardless of soil properties and initial Ni concentrations, over 80% of retained Ni extracted mainly from the residual and the oxidizable fractions suggesting Ni immobilization. Possible mechanisms involved could be surface precipitation as Ni(OH)_2_ and Ni–Al layered double hydroxides and adsorption on the negatively charged surface of cancrinite strongly supported by the FTIR analysis performed on samples of BR and BR amended with 5, 40, and 90 mg Ni L^−1^.

**Supplementary Information:**

The online version contains supplementary material available at 10.1007/s11356-025-36701-z.

## Introduction

The contamination of soils with potentially toxic elements (PTEs) is one of the most serious threats to the natural resources of our planet. Understanding their origin, concentration, and chemical forms in soils is an indispensable prerequisite for their effective management (Zafeiriou et al. 2022). Nickel, classified as one of the PTEs, is the 24th most abundant element on Earth. It naturally exists in various oxidation states, ranging from − 1 to + 4, with Ni^2^⁺ being its most stable state (Begum et al., 2022). From a geochemical perspective, nickel exhibits a strong affinity for sulfur (S) and can also be associated with carbonates, phosphates, and silicates (Alloway 2013). Its presence in soil originates from either lithogenic sources (naturally occurring as a result of parent material weathering) or anthropogenic activities.

Nickel concentrations in soils vary widely, typically ranging from 0.2 to 450 mg kg^−1^, although ultramafic soils can reach levels as high as 5000 mg kg^−1^ (Alloway 2013). As an element, nickel is considered slightly mobile, and its mobility is inversely correlated with soil pH (Boostani et al., 2019).

The main anthropogenic sources of Ni are the industrial effluents from mining, oil refining, mineral processing, electroplating, forging, silver refining, paint formulation, battery manufacturer, and steam electric power plants (Islam et al. 2019). Additionally, long-term use of chemical fertilizers may also cause accumulation (El-Naggar et al. 2021). Nickel enrichment of soil through natural sources, mainly originates from the parent material. Typically, the mineral composition of parent material is linked to soil enrichment with Ni concentrations > 75 mg kg^−1^ in many Mediterranean countries. Soil contamination with Ni from pedogenic processes is mainly associated with ultramafic rocks, such as serpentinites, peridotites, and pyroxenites. However, it is important to note that PTEs derived from natural sources are usually less immobile and toxic compared to those originating from anthropogenic sources (El-Naggar et al. 2021). Tolerance limits for Ni^2+^in water, air, soil, and food are reported as 20 μg/L, 0.025 μg/m^3^, 50 mg/kg d.w., and 132 μg/day, respectively (Islam et al. 2019).

Nickel is considered an essential element for the metabolism of soil microorganisms, plants, and humans. Plants absorb nickel as Ni(H_2_O)_6_^2+^. Its deficiency causes serious imbalances in plant growth, but it is rarely encountered due to the very small quantity required. Conversely, high concentrations are toxic and can result in plant death. Generally, critical levels of toxicity are > 10 mg/g d.w. in sensitive species and > 50 mg/g d.w. in moderately tolerant species (Alloway, 2013).

A variety of solid wastes, including fly ash and bauxite residue, have been evaluated in order to address and regulate heavy metal contamination in soil systems (Feigl et al. 2012; Ruyters et al. 2011; Santona et al. 2006; Wang et al. 2020).

The bauxite residue (BR), often referred to as red mud, is an industrial waste material produced during the extraction of alumina from bauxite through the Bayer process. This solid residue is generated after the bauxite ore is digested with caustic soda for alumina production. The high iron oxide content gives red mud its distinctive reddish-brown color. Additionally, the Bayer process results in red mud being highly alkaline, with a pH range of 10 to 13. As a by-product of alumina manufacturing, bauxite residue is composed of metallic oxides and unreacted minerals from the Bayer process, such as hematite, goethite, gibbsite, and rutile. It also contains compounds formed during the alkali digestion of the ore, including olivine, calcite, sodalite, and sodium aluminosilicate, as well as some residual aluminum oxides (Angelopoulos et al. 2021; Jones and Haynes 2011).

The production of 1 ton of aluminum hydroxide requires 2–3 tons of bauxite and can result in the generation of up to 2.5 tons of this bauxite residue, depending on the characteristics of the ore and the processing conditions (Jones and Haynes 2011). The global production of BR surpassed 150 million tonnes in 2020, and its cumulative total reached 4 billion tonnes by 2022 (Angelopoulos et al. 2024). The vast quantities of red mud produced globally pose significant environmental challenges, as the direct disposal of this waste can lead to the contamination of soil, groundwater, and surface water due to the release of heavy metals, alkali, and other pollutants (Couperthwaite et al. 2014; Mohapatra et al. 2020; Samal et al. 2013).

Currently, BR is commonly utilized as a filler in various construction materials, including cement and cementitious products, bricks, tiles, glasses, glass–ceramics, and the production of geopolymers (Atan et al. 2021; Hertel and Pontikes 2020; Pontikes and Angelopoulos 2013; Ram Kumar and Ramakrishna 2023; Yang et al. 2020). Furthermore, BR has been utilized as a catalyst in diverse industrial processes, including hydrodechlorination, coal hydrogenation, coal/biomass liquefaction, SO_2_ reduction, and methane combustion (Sushil and Batra 2008).

Extracting valuable materials from red mud is another appealing approach, as it contains significant concentrations of metallic components, making it a potential polymetallic resource. Indeed, BR exhibits high iron, aluminum, and titanium content, with the respective oxide and hydroxide forms ranging from 35 to 49%, 11.1–22.6%, and 3.5–15.6% (Agrawal and Dhawan 2021). Also of interest is its high content in rare-earth elements (REEs) which may reach 1700 mg/kg, with up to 160 g/t Sc, 370 ppm Y, 840 ppm Ce, 340 ppm Nd, and up to 2200 ppm Zr (Agrawal and Dhawan 2021; Angelopoulos et al. 2021; Smirnov and Molchanova 1997; Zinoveev et al. 2021). Various techniques have been applied for the extraction of the aforementioned values from BR, which are reviewed in the literature (Akcil et al. 2018; Habibi et al. 2023; Li et al. 2021; Liu et al. 2021; Zinoveev et al. 2021).

The investigation of possible applications of bauxite residue, either through incorporation into the soil profile or as a vertical barrier to create an impermeable layer for heavy metals, is essential. Existing studies provide encouraging indications that bauxite residue could serve as an effective material for these purposes (Rajković et al., 2025). Reviewing relevant literature however, research focusing specifically on the immobilization of various metals in soils amended with BR, examining both their chemical behavior and their equilibrium states, is notably absent. This gap highlights the need for systematic studies to evaluate the potential of bauxite residue incorporation in soil systems to reduce metal mobility.

The aim of this study was to investigate the chemical behavior of nickel adsorption and its subsequent extraction using the Tessier sequential extraction scheme in soils with varying properties, as well as in corresponding soil–bauxite residue (BR) mixtures. The objective was to assess the extent to which soil properties influence the interaction with the inherently stable characteristics of BR at a fixed application rate, and to evaluate the potential of BR as an effective soil amendment for metal immobilization. The findings from this research provide a fundamental basis for determining optimal application rates and for developing management strategies aimed at enhancing soil quality or designing vertical filters and buffer zones within soil systems. Within the broader framework of the circular economy and sustainable land resource management, this study highlights the significance of integrating environmental soil science with the reuse of industrial by-products. Such integration should begin with site-specific strategies and gradually expand to broader spatial scales, including industrial and mining zones, which are often located at the interface of peri-urban and natural or forested land uses. In these contexts, minimizing anthropogenic pollution within the boundaries of each activity is of critical importance.

## Materials and methods

### Soils

Surface soil samples, at depth of 0–20 cm, were collected from six agricultural areas of Attica, Greece, during June 2023, using a stainless-steel auger. The soil samples (named S1–S6) were selected to cover a relatively wide range of typical Greek agricultural soils. The samples were air dried in the laboratory, ground gently, and passed through a 2-mm sieve and routine analyses were carried out. Soil pH and EC (electrical conductivity) were measured electrometrically in a soil/water suspension with a ratio 1:2.5 (w/v), using automated pH meter (Selecta 2000, J.P. Selecta S.A., Barcelona, Spain) and conductivity meter (Selecta 2000, J.P. Selecta S.A., Barcelona, Spain), respectively (Klute and Page 1982). Particle size distribution was performed following the hydrometer method according to Bouyoucos (Bouyoucos 1951). Cation exchange capacity (CEC) was quantified using neutral 1 M sodium acetate solution (Klute and Page 1982). Soil organic matter content was obtained by the Walkley–Black wet oxidation method as described by Nelson and Sommers (1982). Calcium carbonate equivalent (CCE) was measured with a digital calcimeter (FOG L; bd INVENTIONS, Greece) (Bilias and Barbayiannis 2017). Free and amorphous Fe and Mn oxides content were determined after extraction by dithionate-citrate-bicarbonate (DCB) method, and by acid ammonium oxalate method in dark conditions, respectively (Gasparatos et al. 2004, 2006). Pseudo-total Ni concentration was extracted by aqua regia (HCl:HNO_3_ 3:1 v/v) (Epa 3051A) (Agency-US Epa, 2008). The digestion procedure was carried out in a Milestone microwave digestion system (Start D, Milestone, Bergamo, Italy). Ni concentration in the extractants was determined using an atomic adsorption spectrometer (AA240FS, Varian, Middelburg, Netherlands). The main characteristics of the soils are presented in Table 2.

#### Bauxite residue (BR)

Bauxite residue sample was obtained from Mytilineos aluminum plant. BR sample was subjected to overnight drying at 105 °C for moisture removal. Subsequently, particle size distribution was determined on a Malvern Laser Particle Analyzer (Malvern Panalytical Ltd, Malvern, UK). An energy-dispersive X-ray fluorescence instrument Xepos (SPECTRO A.I. GmbH Company) was used for the chemical analysis of the sample. Mineralogical analysis was done to dry ground sample with X-ray powder diffraction (XRPD) analytical technique on a Bruker D8 Advance device (Bruker Corporation, Billerica, MA, USA), under scanning rate of 5° per minute, 2θ range from 10 to 75° using graphite-monochromatized Cu K(alpha) radiation (*λ* = 0.15406 Å). Bauxite residue samples were subjected to IR spectroscopy on PerkinElmer Spectrum 100 FTIR device (ZnSe crystal).

### Chemicals

All the chemical reagents used in this study met or exceeded the standard analytical grade. A stock solution of 1000 mg of Ni per liter was prepared using NiCl_2_ (NiCl_2_ in H_2_O, Titrisol, 1 Amp.) purchased from Merc. All the experimental glassware was immersed for 24 h in 10% (v/v) nitric acid and then rinsed three times with deionized water.

### Nickel sorption by soils and soil-BR mixtures

Soil-BR mixtures were composed by mixing the soil with BR in a rate of 20% (w/w). At present, certain studies have suggested recommended dosage of BR up to 25% (w/w) (Feigl et al. 2012; Lei et al. 2022). The soils and the soil-BR mixtures were equilibrated with different Ni solution concentrations (1, 5, 10, 20, 30, 50, 70, and 90 mg L^−1^). In this study, all sorption experiments were conducted without controlling pH or ionic strength. One gram of adsorbent (soil or soil-BR mixture) was agitated in 50-mL polyethylene centrifuge tube with a 25 mL solution of the appropriate Ni concentration. The samples were shaken in a reciprocal shaker at 120 rpm for 1 h at room temperature (20 ± 2 °C). The selected equilibration time resulted from preliminary equilibration tests. After shaking, the samples were centrifuged in a benchtop centrifuge (K241, Centurion Scientific, Chichester, UK), at 4000 rpm for 10 min and the supernatant was filtered through Whatman filter paper No. 42. The filtrate was analyzed for Ni concentration using atomic adsorption spectrometer (AA240FS, Varian, Middelburg, Netherlands). Calibration was made using standard solutions prepared from stock Ni solution. Reference materials and blank samples were used to verify the accuracy of the measurements. For every ten samples, a control sample was analyzed, and 20% of the samples were reanalyzed to evaluate reproducibility. Simultaneously, Ni adsorption experiments were conducted on BR samples using initial Ni concentrations of 5, 40, and 90 mg kg^−1^, adhering to the same experimental protocol, in preparation for X-ray diffraction (XRD) and Fourier-transform infrared (FTIR) spectroscopy analysis. All adsorption experiments were carried out in duplicate and the mean value was reported. The amount of Ni adsorption at equilibrium ( $${q}_{e})$$ was calculated from the difference between the initial and final Ni concentration, as described by Eq. (1) (Ostovar et al. 2024; Zafeiriou et al. 2023).

1 $${q}_{e}=\frac{\left({C}_{0}-{C}_{e}\right)xV}{m}$$

where ${q}_{e}$ is the adsorption capacity (mg kg^−1^), ${C}_{0}$ is the initial Ni concentration (mg L^−1^), ${C}_{e}$ is the equilibrium Ni concentration (mg L^−1^), *V* is the volume of the solution (L), and *m* is the mass of the adsorbent (g). The adsorption percentage of Ni can be calculated using Eq. (2) (Boulaiche et al., 2019).

2 $$Ads\left(\%\right)=\frac{{C}_{0}-{C}_{e}}{{C}_{0}}\times 100$$

**Fig. 1:**
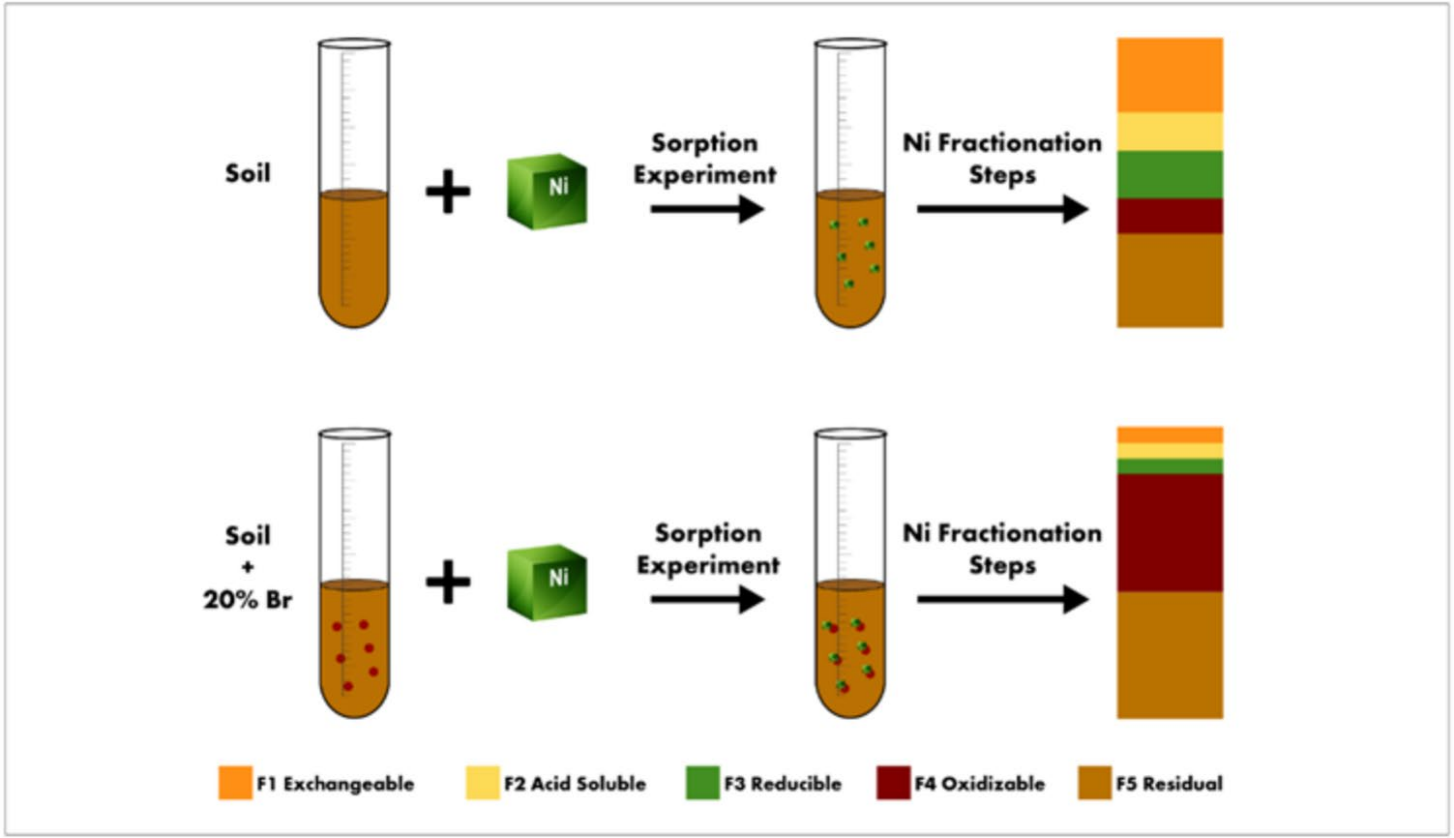
Flow diagram of the experimental design

The distribution coefficient values of adsorption ${(K}_{d})$ were determined by using the following formula (Baghenejad et al. 2016):

3 $${K}_{d}={q}_{e}/{C}_{e}$$

where ${q}_{e}$ is the amount of Ni adsorbed per kilogram of adsorbent (mg kg^−1^) obtained from Eq. (1), and ${C}_{e}$ is the equilibrium Ni concentration (mg L^−1^) in the solution. When evaluated under the same experimental conditions, ${K}_{d}$ is a useful indicator for comparing the sorptive capabilities of various soils (Ding et al. 2018; Kumar et al. 2020; Shaheen et al. 2013) and is required for modeling heavy metal transport into the environment (Fig. 1).

### Sequential extraction of nickel

Immediately after sorption experiments, a modified five-step Tessier sequential extraction protocol (Tessier et al. 1979) was applied to all Ni-contaminated soils and soil-BR mixtures, aiming to identify Ni speciation in the tested absorbents (Table 1). Compared to the sequential extraction, fractionation using single extractants is less laborious and demands less skilled personnel but most probably increases the variability of the results since different soil samples used for every single extraction (Tack and Verloo 1999). According to the modified five-step Tessier sequential extraction procedure, the five operationally defined Ni fractions are as follows: exchangeable (F1), carbonate bound (F2), reducible (F3), oxidizable (F4), and residual (F5). The procedure described above was followed for shaking, centrifugation, and filtration of the extractants after each extraction step, whereas any deviations of the absorbents weight from the initial 1 g were taken into account in the calculation of the results. The extraction procedure was carried out in duplicate and Ni concentration in the solutions was determined by atomic adsorption spectrometer.

**Table 1:** Sequential extraction scheme (1 g of solid)

Step	Fraction	Reagents	Procedure
1	**F1** Exchangeable	8 mL 1 M MgCl_2_ (pH = 7)	1-h shaking 120 rpm room temperature
2	**F2** Carbonate bound	8 mL 0.11 M NaHCO_3_ (pH = 5.0 with HOAc)	5-h shaking 120 rpm room temperature
3	**F3** Reducible bound to Fe/Mn oxides	20 mL 0.04 M NH_2_OH·HCl in 25% (v/v) HOAc (pH = 2.0)	6-h shaking 120 rpm *T* = 96 ± 0.5 °C
4	**F4** Oxidizable bound to organic matter and sulfides	3 mL 0.02 M HNO_3_ + 5 mL 30% H_2_O_2_ (pH = 2.0 with HNO_3_) 3 mL H_2_O_2_ (pH = 2) 5 mL 3.2 M NH_4_OAc in 20% (v/v) HNO_3_ and the mixture diluted to 20 mL	1-h digestion room temperature occasionally shaking 1-h digestion *T* = 85 °C occasionally shaking 3-h digestion *T* = 85 °C occasionally shaking 30-min shaking 120 rpm room temperature
5	**F5** Residual	9 mL 37% HCl + 3 mL 65% HNO_3_ (USEPA method 3051 A)	Digested in a microwave 15 min until *T* = 200 °C 15 min at 200 °C

Numerous researchers applied Tessier sequential extraction in soils, BR, and other solid wastes to investigate the existing forms of heavy metals (He and Kasina 2023; Okoro and Fatoki 2012; Qi et al. 2020). Tessier protocol, as with other sequential extraction schemes, is operationally defined and inherently unreliable to an extent as it is subject to phase carryover from incomplete extraction. However, sequential extraction is permissible if all samples are treated identically, allowing for the identification of metal phase trends.

Nickel recovery rate (RR_Ni_%), which was calculated as the sum of Ni concentration in the five operational fractions divided by the pseudo-total Ni concentration (all in units of mg kg^−1^) (Eq. 4), was used to confirm the accuracy of the Tessier sequential extraction results.

4 $${\text{RR}}_{\text{Ni}}\text{\%}=\frac{\text{F}1+\text{F}2+\text{F}3+\text{F}4+\text{F}5}{{T}_{\text{Ni}}}\text{\%}$$

where ${\text{RR}}_{\text{Ni}}\text{\%}$ is the recovery rate of Ni (%); F1,…F5 is the Ni concentration in the five fractions (mg kg^−1^); and ${T}_{\text{Ni}}$ is the pseudo-total Ni concentration (mg kg^−1^).

In this study, Ni mobility and bio-availability in the examined soils and soil-BR mixtures were estimated by using the mobility factor (*M*_*F*_), a measure of the degree of metal binding that is frequently used to quantify heavy metal pollution in sediments and soils (Klik et al. 2020).

According to Kabala and Singh (2001), the mobility factor (*M*_*F*_) of Ni was calculated from the results of the sequential extraction as follows:

5 $${M}_{F}=\frac{\text{F}1+\text{F}2}{\text{F}1+\text{F}2+\text{F}3+\text{F}4+\text{F}5}\text{\%}$$

where F1, F2…F5 are the concentration of the specified metal in each fraction of the sequential extraction that was used. Less than 1% of heavy metals in F1 + F2 is considered to pose no risk (NR), 1% to 10% indicates low risk (LR), 10% to 30% medium risk (MR), 30% to 50% high risk (HR), and > 50% indicates very high risk (VHR) (Gusiatin & Klimiuk 2012; Sundaray et al. 2011). However, high F1 and F2 concentrations indicate potential hazard.

The relative binding intensity (${I}_{R}$) may be used to assess metal stability and redistribution in soil (Han et al. 2003). ${I}_{R}$ was calculated as follows:

6 $${I}_{R}=\frac{\sum_{i=1}^{25}\left({F}_{i}x{(i)}^{n}\right)}{{K}^{n}}$$

where *i* represents the number of the extraction step (*i* = 1, 2, 3, 4, and 5), *k* value is 5 for the five-step Tessier method, ${F}_{i}$ is the fractional percentage amount of Ni, and *n* integer (mainly 1 or 2). It is optional to choose *n.* When *n* equals 2 indicates that the ions’ binding strengths are intensifying as the extraction and calculation stages proceed (Piri et al. 2021).

### Adsorption isotherms

Sorption equilibrium isotherms can provide the fundamental physicochemical information for determining the suitability of an adsorption system. The isotherm models describe the surface characteristics and affinity of the adsorbent material, as well as the relationship between the amount of adsorbate absorbed by the adsorbent and the concentration of adsorbate in the solution. Many adsorption isotherms can be used to determine the aqueous-phase adsorption process, but the Langmuir (1918) and Freundlich (1906) isotherms are the most commonly applied.

Assuming monolayer coverage of the surface and negligible interaction forces between adsorbed atoms, the Langmuir adsorption isotherm (7) is expressed as follows:

7 $$\frac{{C}_{e}}{{q}_{e}}=\frac{1}{{b}_{L}{q}_{m}}+\frac{{C}_{e}}{{q}_{m}}$$

where ${q}_{m}$ (mg g^−1^) is the maximum adsorption capacity of the adsorbent for Ni, and ${b}_{L}$ (L g^−1^) is the Langmuir equilibrium or affinity constant related to adsorption energy (binding site affinity). ${q}_{m}$ and ${b}_{L}$ were calculated using the slope and intercept of the ${C}_{e}$/${q}_{e}$ versus ${C}_{e}$ linear graph.

In addition, the dimensionless constant ${R}_{L}$ (Eq. 8), also known as the separation factor or equilibrium parameter (Vargas et al., 2011), was calculated as follows (Nnaji et al. 2021):

8 $${R}_{L}=\frac{1}{1+{b}_{L}x{C}_{i}}$$

where ${C}_{i}$ is the initial Ni concentration (mg L^−1^).

$R_L$ is used to determine the viability of the sorption process in the studied system, with sorption being unfavorable, linear, favorable, or irreversible, respectively, if $R_L>1,R_L\;=\;1,\;R_L\;<1,\;or\;R_L=0$ 

Freundlich adsorption isotherm is utilized to describe adsorption on heterogeneous surfaces under the assumption that multilayer adsorption is possible (Foo and Hameed 2010). It is described by an empirical equation whose linear form is expressed as follows:

9 $${\text{log}q}_{e}=\text{log}{K}_{f}+\left(\frac{1}{n}\right)\text{log}{C}_{e}$$

where ${K}_{f}$ is the Freundlich constant (mg g^−1^) related to the adsorption capacity, and *n* is an empirical parameter, referring to the adsorption intensity. The values of ${K}_{f}$ and *n* were calculated from the slope and intercept of the plot ${\text{log}q}_{e}$ versus $\text{log}{C}_{e}$. If the value of *n* is in the range 1 < *n* < 10, the adsorption is favorable.

## Results and discussion

### Characterization of soils

Table 2 presents the main physicochemical parameters, and the total Ni concentration of the soils used in this study. The pH values range from 5.41 (S4) to 7.73 (S1), covering a relatively wide band of soil pH values. Clay content varies between 19.00% (S4) and 37.00% (S3) and the organic carbon content is low in all soils as it ranges from 1.02% (S5) to 1.86% (S3), portrays the oxidizing conditions that prevail under Mediterranean climatic conditions (Gasparatos et al. 2011). CEC values ranged from 13.39 cmol_c_ kg^−1^ (S4) to 30.26 cmol_c_ kg^−1^ (S3). S1 and S6 have the highest percentage of CCE (3.8% and 2.4%, respectively). Pseudo-total Ni concentration in the studied soils ranged from 36.50 to 69.30 mg kg^−1^. The amounts of dithionite and oxalate extractable iron and manganese, expressed as grams of Fe and Mn oxides per 100 g of dry soil, showed significant variability pointing to different pedogenetic conditions and soil evolution stages.

**Table 2:** Selected physicochemical properties of the soils used in this study

Soil properties	Soil					
S1	S2	S3	S4	S5	S6	
Clay (%)	25.0	27.0	37.0	19.0	21.1	23.1
Silt (%)	34.0	20.0	20.0	28.0	37.5	38.4
Sand (%)	41.0	53.0	43.0	53.0	41.5	38.5
Texture	L	SCL	CL	SL	L	L
pH (1:1)	7.73	6.03	6.90	5.41	7.58	7.56
O.C. (%)	1.02	1.53	1.86	1.11	0.99	1.56
CCE (%)	3.81	0.00	0.00	0.00	0.62	2.42
CEC (cmol_c_ kg^−1^)	13.48	22.17	30.26	13.39	18.78	18.35
Fe_d_ (%)^a^	2.69	2.91	2.84	2.19	1.13	0.95
Fe_o_ (%)^b^	0.16	0.33	0.37	0.14	0.09	0.12
Mn_d_ (%)^c^	0.11	0.17	0.15	0.19	0.04	0.04
Mn_o_ (%)^d^	0.05	0.21	0.18	0.51	0.04	0.03
Ni (mg kg^−1^)	39.60	56.05	56.60	36.50	69.30	56.80

*CCE* calcium carbonate equivalent

^a^free iron oxides

^b^amorphous iron oxides

^c^free manganese oxides

^d^amorphous manganese oxides

### Characterization of BR

The chemical composition of BR is illustrated in Table 3. The BR sample showed high content in iron oxide (46.70%) as well as in residual aluminum (15.40%), low sodium content (3.94%), and a noticeable present of calcium oxide (9.46%).

**Table 3:** Chemical composition of BR sample (XRF)

Oxide	Na_2_O	MgO	Al_2_O_3_	SiO_2_	K_2_O	CaO	TiO_2_	Cr_2_O_3_	Fe_2_O_3_	NiO	LOI	Other
(%)	3.94	0.18	15.40	8.27	0.13	9.46	6.16	0.29	46.70	0.12	8.29	1.06

**Fig. 2:**
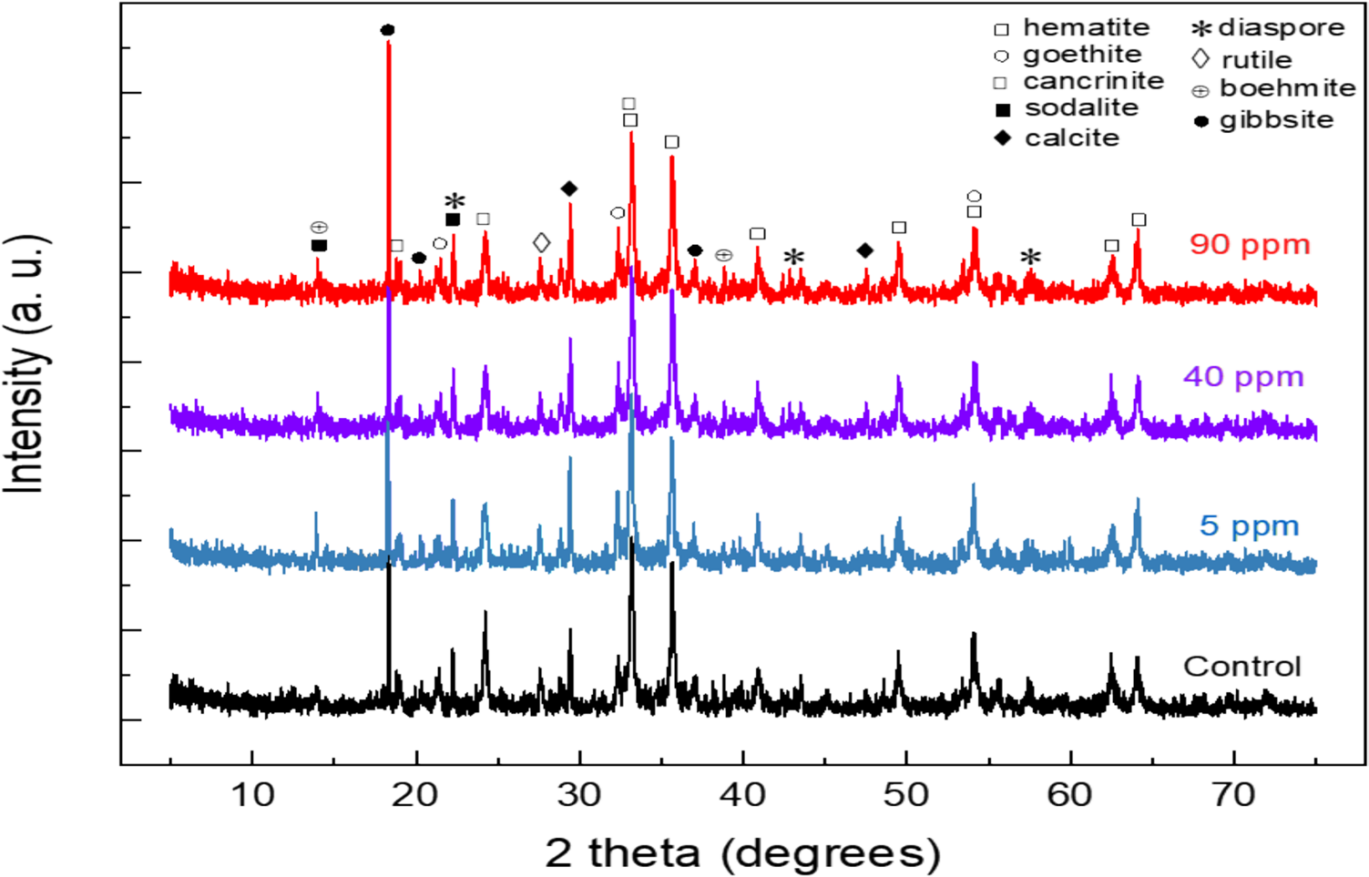
X-ray diffractogram for bauxite residue (control) and bauxite residue sample after Ni sorption at various initial metal concentrations

Goethite, hematite, and rutile, which is the phase hosting the titanium, are the main iron phases in the bauxite residue (BR), and calcium is present as calcite (Fig. 2, control). Diaspore and boehmite are the main Al phases. Other identified minerals were sodalite and cancrinite (Angelopoulos et al. 2021). In the same figure (Fig. 2), XRD patterns of BR after sorption of different Ni concentrations (5, 40, and 90 mg L^−1^) are presented. The insignificant differences between the diffractograms indicate that the sorption of Ni on BR did not lead to an apparent structural or mineralogical composition alteration in the BR. As for the size distribution of BR, the D10, D50, and D90 of BR were found as 1.1 μm, 3.7 μm, and 36.2 μm, respectively, which are the typical values for the Greek BR (Angelopoulos et al. 2021) (Angelopoulos et al. 2024).

### FTIR spectra of bauxite residue before and after nickel sorption

**Fig. 3:**
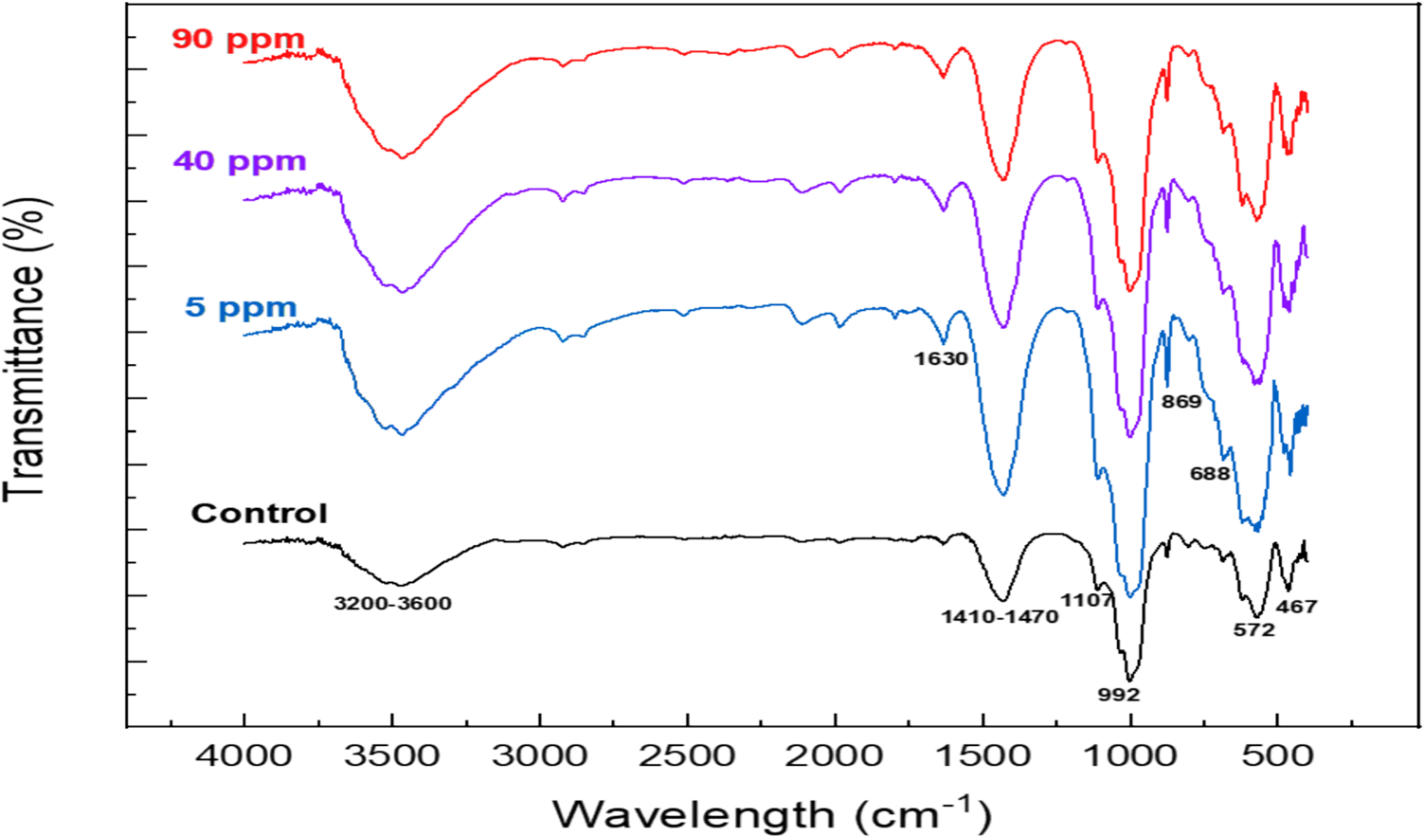
FTIR spectra of bauxite residue (control) and bauxite residue sample after Ni sorption at various initial metal concentrations

The infrared spectrum obtained for the BR before and after the sorption of 5, 40, and 90 mg Ni L^−1^ is displayed in Fig. 3. The wide peak at the 3200–3600 cm^−1^region is attributed to the asymmetric and symmetric vibrational modes of O–H and is representative of mineral hydrates and metal hydroxides (Arogundade et al. 2021; Castaldi et al. 2008; Singh et al. 2019). The peak at around 1630 cm^−1^ is caused by the bending of water molecules (H–O-H) that are present in goethite (Arogundade et al. 2021; Castaldi et al. 2008). Mucsi et al. (2021) identified the presence of a band between 1630 and 1650 cm^−1^ as molecular water in cancrinite. Carbonates’ presence is confirmed by a relatively wide band at 1410–1470 cm^−1^ and strong asymmetric C-O stretching vibrations centered at 1435 cm^−1^ formed by calcite (Castaldi et al. 2008; Mucsi et al. 2021). Also, C-O bending causes the band at 869 cm^−1^ (Mucsi et al. 2021). Yadan et al. (2010) identified peak at around 1425 cm^−1^ on Ni-loaded gravel and they claimed that it proved the interaction between functional groups (–O–Si = O, SO_2_OH) and metal ions. Interestingly, here, the peak appears significantly sharper for doped bauxite residue samples, which can be attributed to the Ni adsorption on bauxite residue. The peak identified at around 1107 cm^−1^ can be due to the asymmetric stretching of Si–O-Al network or the O-Fe–O of goethite (Castaldi et al. 2008; Mucsi et al. 2021). The anti-symmetric stretching vibration of silica Si–O is found in 992 cm^−1^ (Arogundade et al. 2021). At around 688 cm^−1^, BR shows common peaks due to the symmetric stretch of the Si–O-Al framework (Castaldi et al. 2008; Mucsi et al. 2021). Sodalite and cancrinite, being very similar in terms of their structural features, show common peaks in the region 400–500 cm^−1^, centered at 467 cm^−1^ due to the T-O bend (where T = Si or Al), and in the region 560–630 cm^−1^ due to 4- or 6-membered ring vibrations of SiO_4_ or AlO_4_ tetrahedra (Barnes et al. 1999). The performed FTIR analysis was in line with the chemical composition and XRD spectra of BR (Table 3 and Fig. 2).

Ni sorption on BR produced noticeable alterations in FTIR spectra. The sharper form of the absorption bands at 3200–3600, 1410–1470, and 992 cm^−1^ may be attributed to hydrogen bond formation and precipitation of Ni(OH)_2_ on the BR surface, Ni sorption on carbonate phase, and on Si bearing minerals respectively (Smičiklas et al. 2013). Another important shift observed for the 572 cm^−1^ absorption band indicating Ni adsorption on cancrinite. Bauxite residue contains high quantities of cancrinite (Na_6_[Ca_2_(CO_3_)_2_Al_6_Si_6_O_24_]·2H_2_O), an aluminosilicate mineral with sodium and calcium that belongs to the feldspathoid group. Cancrinite shows high sorption capacity for metals due to its high negative surface charge. Metal ions absorbed on external surfaces while ions of compatible ionic radius incorporated into the cages and channels of the internal structure (Santona et al. 2006).

### Nickel sorption on soils and soil-BR mixtures

**Fig. 4:**
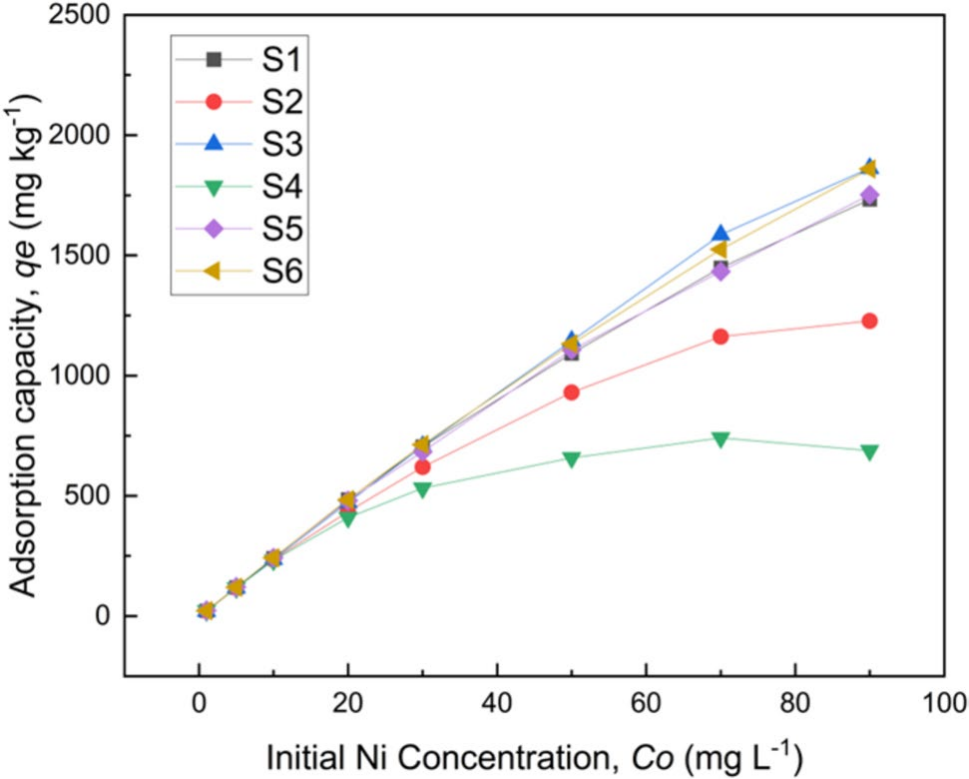
Amount of Ni sorbed on soils against its initial solution concentration

Nickel sorption capacity (*q*_*e*_) of the six soils vs. Ni initial concentration in the solution (*C*_*0*_) is presented in Fig. 4. Ni sorption capacity increased almost linearly over the whole range of the tested *C*_*0*_ for the samples S1, S3, S5, and S6, and ranged between 22.33–1733.00, 20.43–1863.00, 23.08–1752.50, and 22.58–1859.50 mg Ni kg^−1^, respectively. Different sorption patterns were observed for soils S2 and S4. In particular, *q*_*e*_ of S2 was lower than previous mentioned soils, reaching 1227.50 mg kg^−1^ at the highest Ni solution concentration, whereas S4 was progressively saturated and showed a maximum *q*_*e*_ of only 741.50 mg kg^−1^ when the initial solution Ni concentration was 70 mg L^−1^. The higher Ni sorption capacity of the alkaline soils (S1, S3, S5, S6) compared to the more acid soils (S2, S4), is in accordance with the results reported by many researchers. Ramachandran and D’Souza (2013), studying adsorption behavior of Ni on many tropical soils, reported that soil adsorption capacity for Ni increased as pH increased, whereas the opposite was shown to be true for adsorption rate. Gollia et al. (2023), studying metal adsorption on polluted urban soils with different pH values, concluded that the retention of metals in alkaline soils was much higher than the acidic soils. Apart from the low pH, soil S4 has the lowest clay content and low C.E.C., factors that may limit its sorption capacity. In contrast, S3 with the highest clay and organic carbon content showed the highest adsorption capacity among the six soils. This result is consistent with the findings of Covelo et al. (2004), who demonstrated that the soils with the highest organic matter, oxide, and clay content are also those that adsorb the greatest amount of metals.

The obtained equilibrium data for Ni adsorption on the studied soils were well described by the linear regression of Langmuir and Freundlich isotherm models. The results are illustrated in Table 4.

**Table 4:** Langmuir and Freundlich isotherm constants for the adsorption of Ni on soils

Soils	max *q*_*e*_ (mg g^−1^)	Langmuir				Freundlich		
		*q*_*m*_ (mg g^−1^)	*b*_*L*_ (L g^−1^)	*R*^2^	*R*_*L*_	*n*	*K*_*f*_	*R*^2^
S1	1.73	1.91	0.29	0.97***	0.04	1.50	12.46	0.87**
S2	1.23	1.31	0.22	0.98***	0.05	1.64	9.72	0.88**
S3	1.86	2.44	0.21	0.96***	0.05	1.24	12.49	0.95***
S4	0.69	0.67	0.68	0.98***	0.02	2.16	8.89	0.80*
S5	1.75	1.93	0.28	0.97***	0.04	1.52	12.45	0.89**
S6	1.86	2.13	0.31	0.97***	0.03	1.42	13.30	0.91**

*p < 0.05, **p < 0.01, ***p < 0.001

The high regression coefficient (*R*2 > 0.96) indicates a strong correlation between the experimental data and the Langmuir isotherm model, suggesting that the sorption of Ni onto the soil surfaces predominantly follows monolayer adsorption behavior. This conclusion is further supported by the close agreement between the experimentally determined (max* q*_*e*_) and theoretically calculated (*q*_*m*_) maximum sorption capacities, which represents the monolayer coverage of the sorbent by the sorbate.

Parameters *n* and* K*_*f*_ were derived from fitting the experimental data to the Freundlich model. The *n* values for all soil samples ranged from 1.24 to 2.16, indicating favorable sorption conditions and suggesting strong interactions between Ni ions and the heterogeneous binding sites present on the soil surfaces. However, despite the suitability of the Freundlich model in describing sorption on heterogeneous surfaces, the higher *R*2 values obtained for the Langmuir model confirm its greater appropriateness in representing the experimental sorption behavior of Ni in the tested soils.

**Fig. 5:**
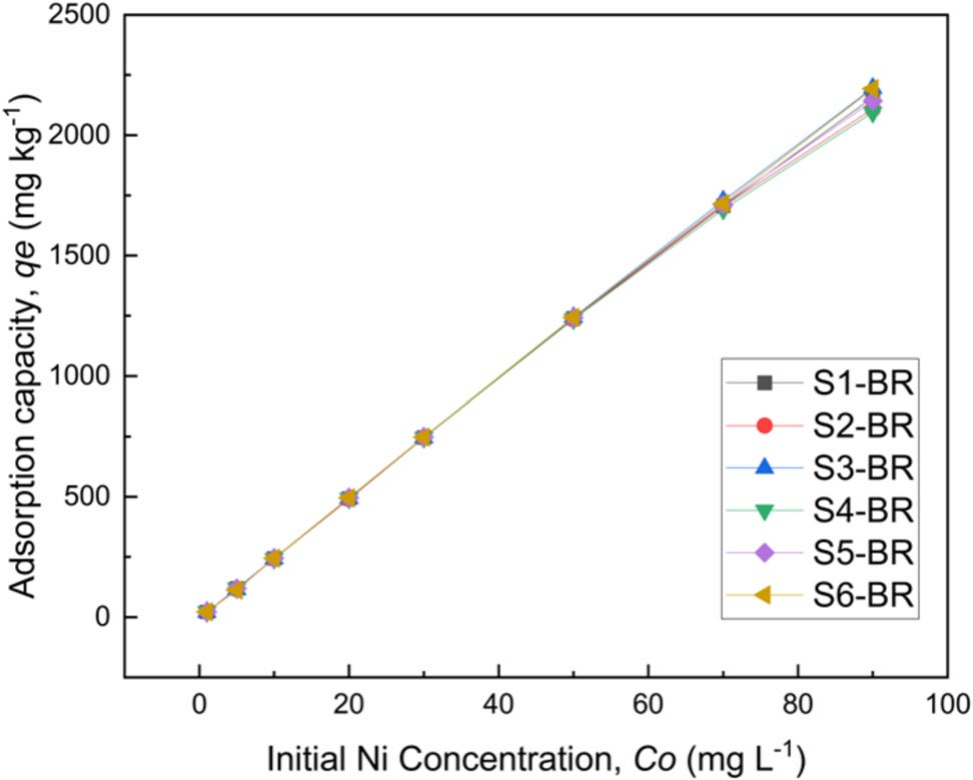
Amount of Ni sorbed on soil-BR mixtures versus its initial solution concentration

In the presence of BR, Ni adsorption (*q*_*e*_) on all soils increased gradually over the whole range of the studied metal concentrations (Fig. 5). The effect of BR on Ni retention was especially impressive in the case of S4-BR mixture, where *q*_*e*_ increased from 688.50 mg kg^−1^ on soil alone to 2094.73 mg kg^−1^ on soil-BR mixture, at the highest *C*_*0*_.

Neither Langmuir nor Freundlich adsorption models were able to describe the sorption data of Ni on the soil-BR mixtures, since over the tested Ni solution concentrations, no sorption maxima was reached. Though the experimental data show a progressive saturation of sorption sites on soil-BR mixtures, the C-type isotherms presented in Fig. 5 indicate a constant proportional affinity of Ni for the soil-BR mixtures. Considering that C-type isotherm is noticed at low range of adsorbate concentration, it can be assumed that, depending on soil properties and under the selected experimental conditions, the sorption capacity of soil-BR mixtures is much higher.

Due to the alkaline nature of red mud (pH = 10.68), the incorporation of 20% (w/w) BR into soils rapidly increased soil pH values that maintained over the whole experimental period. The respective pH values for S1, S2, S3, S4, S5, and S6 mixtures were 9.31, 8.11, 8.50, 8.19, 9.10, and 9.12. Compared to soils, the higher Ni sorption on soil-BR mixtures can be attributed to the increased pH values. Gräfe and his co-workers (2011) found that sequential washings of BR decreased solid weight but did not affect pH, Na^+^, Al(OH)_4_^−^, CO_3_^2−^, or OH^−^ concentrations in solution. Their experiment showed that alkaline materials buffered the pH of the residue solution, which remained stable until the solids were entirely dissolved, and their reaction products were eliminated. An increase in soil pH from 0.1 to 4.8 pH units has been reported by Hua et al. (2017) whereas Lee et al. (2011) showed that the addition of 5% w/w BR raised soil pH from 5.22 to 9.4. Many authors (Brown et al. 2005; Gray et al. 2006; Lombi et al. 2002) report that soil pH increased between 0.7 and 2.3 units after the addition of 2% bauxite residue. Furthermore, the findings of many studies support the direct effect of raising pH on increased metal adsorption in soils (Raghupathi and Vasuki, 1993; Gomes et al. 2001; Ramachandran and D’Souza 2013). The higher metal sorption has been attributed to the weaker metal ions competition with H^+^, the hydrolysis state of the metals in the solution, and the increase of pH-dependent sites on soil colloid surfaces. At higher alkaline pH levels, Ni sorption is enhanced by the precipitation of Ni^2^⁺ and NiOH⁺ with hydroxides. It has been reported (Gupta and Bhattacharyya, 2006; Roberts et al. 1999) that the addition of Ni to soils results in the formation of mixed Ni–Al hydroxide surface precipitates within 15 min at pH ≥ 7.5. They observed that if sources of soluble Al were present in the sorbent structure, a Ni–Al layered double hydroxide developed and suggested that precipitate formation is an essential process in early response times and dominates Ni absorption at longer reaction times. In contrast, Ni sorption on non-Al-bearing minerals (talc, silica) produced a-Ni(OH)_2_. In soils where easily soluble Ni forms are added rapidly, mixed Ni–Al hydroxide are less stable and precipitation forms of Ni have characteristics more similar to Ni(OH)_2_, particularly when sources of available Al are limited (Peltier et al. 2010).

**Fig. 6:**
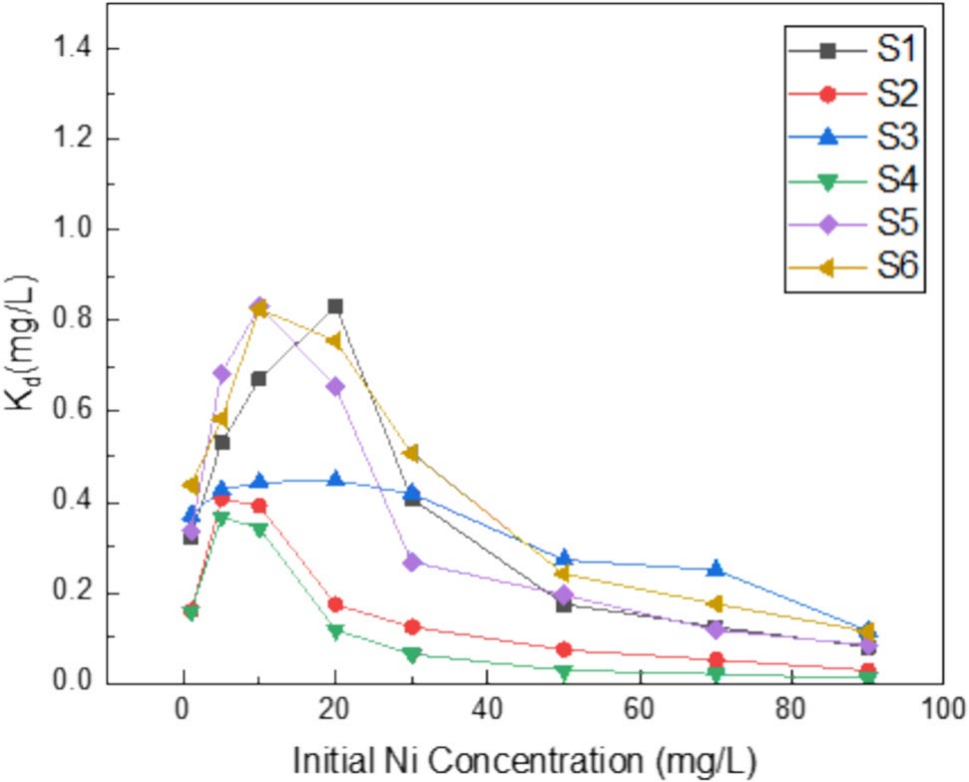
Distribution coefficient ${(K}_{d})$ of soils as a function of initial Ni concentration (*C*_*0*_)

**Fig. 7:**
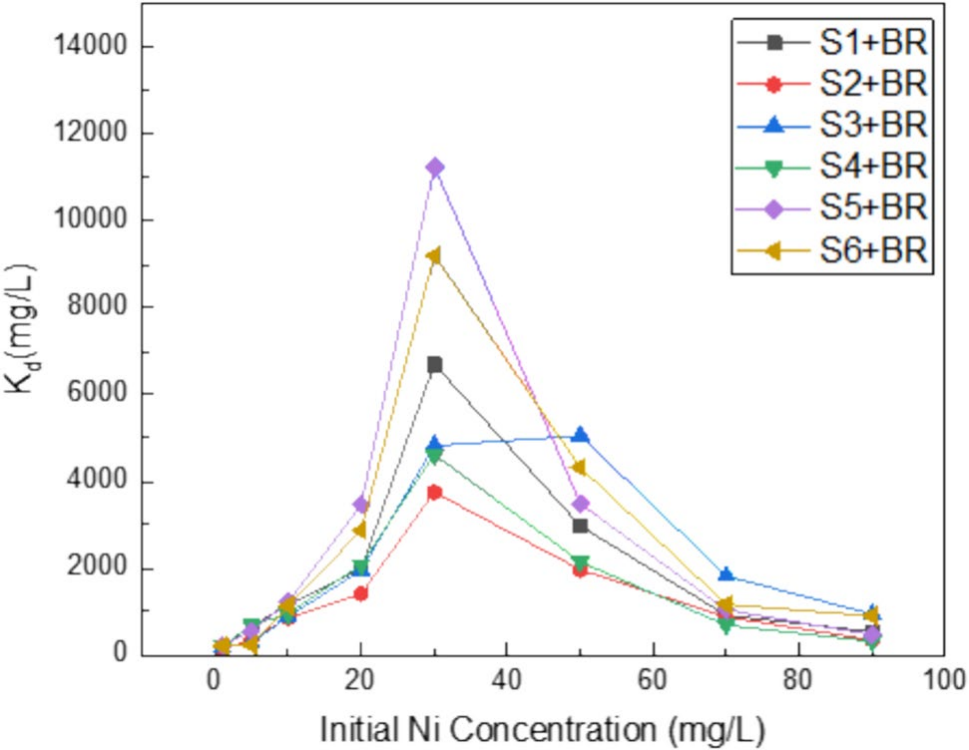
Distribution coefficient ${(K}_{d})$ of soil-BR mixtures as a function of initial Ni concentration (*C*_*0*_)

Figures 6 and 7 portray the relationship between the solid–liquid distribution coefficient ${K}_{d}$ for the studied soils or the corresponding soil-BR mixtures and the initial solution concentration (*C*_*0*_), respectively. The equilibrium between solid and liquid phases regulates the mobility and toxicity of metals in soils. The greater the ${K}_{d}$ value, the easier it is for Ni to be retained in soil by sorption; conversely, the lower the ${K}_{d}$ value, the more Ni is present in the soil solution (Sauve et al. 2003). A higher total metal concentration typically results in a lower proportion of metals being adsorbed because potential adsorption sites are occupied in decreasing order of affinity. The more the soil becomes saturated with cations, the less metal-affine the residual sites become. It is expected then the resulting ${K}_{d}$ values to decrease as the initial metal concentration (*C*_*0*_) increases.

In the six studied soils, the ${K}_{d}$ increased at lower Ni solution concentrations and then dropped gradually at higher Ni solution concentrations (Fig. 6). Over the whole range of added Ni concentrations, the ${K}_{d}$ values were lower for the more acid soils than for the more alkaline soils, in accordance with the pH effect on sorption capacity of the soils. Specifically, when *C*_*0*_ was 5 mg L^−1^, the ${K}_{d}$ value reached its maximum value in the acid soils (S2, S4) and dropped steeply after the 10 mg Ni L^−1^ concentration. The maximum ${K}_{d}$ value for S5 and S6 was reached at *C*_*0*_ 10 mg L^−1^, while for S1 at 20 mg L^−1^. For S3, the ${K}_{d}$ value remained almost constant up to *C*_*0*_ 30 mg L^−1^ and then decreased at higher Ni concentrations.

From Fig. 7, it is apparent that the incorporation of BR in soils extremely increased maximum ${K}_{d}$ values by approximately 10,000 times, clearly pointing to different sorption mechanisms involved as high selectivity sorption sites with stronger bonding energies and the formation of new Ni species. Maximum ${K}_{d}$ values were observed at *C*_*0*_ 30 mg L^−1^ Ni except for S3-BR mixture that maximum ${K}_{d}$ value was observed at *C*_*0*_ 50 mg L^−1^.

### Ni fractionation in soils and soil-BR mixtures

**Fig. 8:**
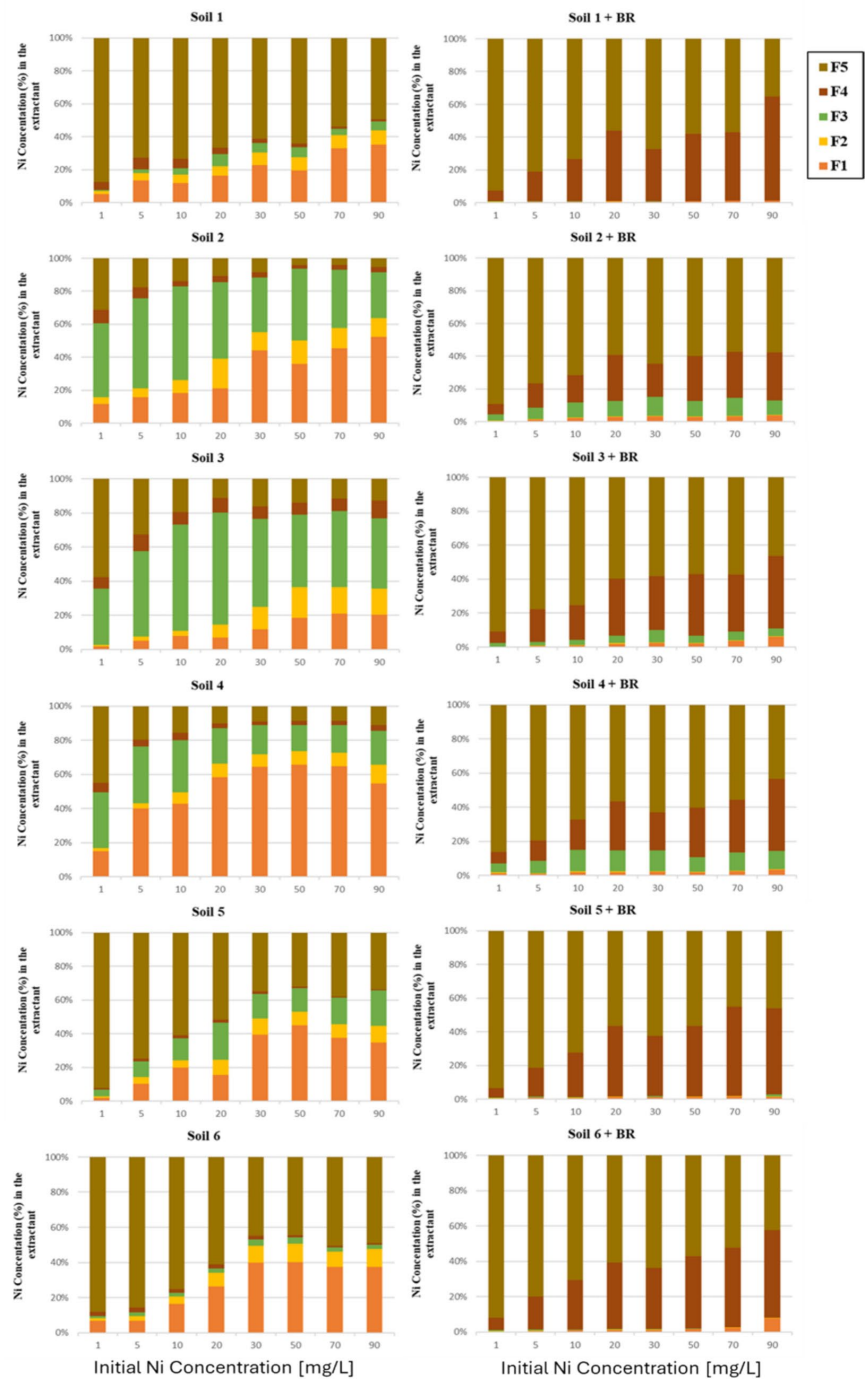
The relative percentage of Ni fractions in the studied soils and the corresponding soil (left)-BR mixtures (right)

Understanding metal speciation in soils is key to predicting heavy metal mobility and bioavailability. Sequential chemical extraction, using various reagents and conditions, identifies metal binding sites and phase associations (Yuan et al., 2004), enhancing knowledge of geochemical processes and environmental risks. To analyze Ni speciation, contaminated samples from batch sorption experiments underwent a modified Tessier sequential extraction (Tessier et al. 1979). Ni recovery rates were within the US EPA’s acceptable range of 75–125% in 98% of cases (U.S. Epa 2010). Figure 8 shows Ni distribution across soil and soil-BR mixtures, expressed as a percentage of pseudo-total Ni concentration.

### Soils

As it presented in Fig. 8 (diagrams on the left), the contribution of the five fractions to total Ni concentration that was retained by each soil differs among the studied samples and is mainly controlled by the soil properties (Antoniadis and Tsadilas, 2007; Campillo-Cora et al., 2021) and the amount of freshly added Ni. At the lowest *C*_*0*_ (1 mg Ni L^−1^), the predominant fraction is the residual for all soils except for the most acidic soil S4, in which the reducible fraction predominates. In all soils, increasing *C*_*0*_ resulted in increased Ni concentration in the exchangeable fraction while Ni presence in the residual fraction reduced as additional metal went into less stable forms (Nyamangara, 1998). At higher *C*_*0*_, the exchangeable fraction represented almost 50% of the total Ni concentration and even reached 65% in the more acidic soil S4.

### Soil-BR mixtures

Nickel fractionation showed a completely different pattern in the soil-BR mixtures compared to the soils (Fig. 8). Most of the metal was recovered in the residual and oxidizable fraction, while the exchangeable and the acid soluble, which are the more mobile metal fractions in soils, decreased at all added Ni concentrations and for all soil-BR mixtures. The exchangeable fraction was nearly vanished and in most cases was below 2% of the total Ni amount. The incorporation of BR in soils greatly altered Ni bonding on chemically active fractions and, depending on the soil, led to Ni immobilization or to the substantial reduction of Ni mobility in the soil environment. Santona et al. (2006) tested metal immobilization by red mud and report that only low amounts of the adsorbed Pb, Cd, and Zn were found in the water-soluble and exchangeable fractions, because most of the added metal quantity was tightly bound and would not be expected to be released under natural conditions. Similar results are reported by Feigl et al. (2012) who found that red mud application to agricultural soils decreased the mobility of metals. Pavel et al. (2015) demonstrated that applying bauxite residue to heavily contaminated soils raised the pH of the soil by about 2 units and decreased the mobile fractions of Zn, Cd, and Pb whereas the metal concentration bound to Fe oxides increased. However, in the present study, the reducible Ni concentration, which is likely to be associated with Fe–Mn oxides, was not the predominant fraction. In adsorption matrices with high Fe content, as the BR used in this study, hydroxylamine is considered as not capable to fully dissolve Fe–Mn oxides, especially the well-crystallized phases, and therefore dissolves only amorphous Fe and Mn oxides (Gleyzes et al. 2002; Kalyvas et al. 2018). Thus, some of Ni bound to Fe–Mn oxides that hydroxylamine failed to extract was probably incorporated to the residual fraction of the Tessier scheme. Various mechanisms may be involved in Ni immobilization in the studied soil-BR mixtures may have occurred as surface precipitation that according to Collins et al. (2014) is the primary procedure or like diffusion in the solid-water interface and migration into the pores of the solid as supported by Hamon et al. (2002). At high pH values, as those of soil-BR mixtures, the readily mobile fraction of metals was reduced probably because of metal adsorption on negatively charged soil surfaces and metal precipitation in the form of hydroxides and carbonates. However, pH change is not the only factor influencing metal distribution in active chemical fractions for the studied soil-BR mixtures. The mineralogy of BR as well as its Al, Fe, and Ca content plays a significant role in the immobilization of metals (Gräfe et al. 2011; Zhu et al. 2016).

The main Al-phases identified in the bauxite residue used in this study were Al-geothite, diaspore, and boehmite whereas hematite was the main iron phase (Table 3, Fig. 1); all of them may be involved in Ni immobilization in soil-BR mixtures with the availability of the substrate cations, especially aluminum. In alkaline conditions precipitates of Ni(OH)_2_ and Ni–Al-DLH (double layered hydroxides) are the main mechanisms of Ni fixation onto soil constituents (Qiang et al. 2017). The results of Roberts et al. (1999) showed that the Ni–Al LDH precipitate can form within 15 min at pH 7.5. Such Ni-LDH phases show high resistance against dissolution even under acidic conditions (Voegelin and Kretzschmar 2005), which is very important to evaluate the bioavailability and toxicity of Ni in the environment. Interestingly, the results of the sequential extraction that showed decreasing residual Ni fractions of soil-BR mixtures at higher Ni concentrations are in accordance with the modifications of the FTIR spectrum that were stronger at the lower concentration of added Ni.

Overall, metal precipitation processes may explain the high degree of irreversible Ni binding on soil-BR mixtures. Future experiments with the use of X-ray adsorption spectroscopy (XAS) could provide a better understanding of Ni immobilization process in soil-BR mixtures.

### Effect of bauxite residue on nickel binding intensity and mobility in the soils

#### Relative binding intensity

Considering that the range of ${I}_{R}$ is narrow, taking values between 0.04 and 1, ${I}_{R}$ can effectively summarize the sequential extraction results of the soils and soil-BR mixtures. In the present study, value *n* = 2 was preferred for ${I}_{R}$ calculation because the ion’s binding strength is intensifying as the Tessier extraction procedure proceeds. According to Tang et al. (2017) and Yang and Hodson (2019), the ${I}_{R}$ value closed to 1.0 implies a high percentage of the metal being tightly bound in the residual fraction, suggesting a low potential environmental concern. In contrast, an extremely low (almost zero) value of ${I}_{R}$ indicates that a significant portion of the metal is present in the exchangeable fraction, indicating a comparatively high environmental threat. As was expected, ${I}_{R}$ values decreased as the initial amount of Ni added in the soils and the soil-BR mixtures increased and were much higher for the soil-BR mixtures (Table 5). Both the considerably higher ${I}_{R}$ values calculated for the soil-BR mixtures compared to soils and the much smaller range between the highest and the lowest ${I}_{R}$ value for the soil-BR mixtures than for the soils strongly support that the addition of BR increased Ni fixed to more stable fractions reducing its possible mobility and availability. However, the more pronounced effect is that the addition of BR in the S2, S3, and S4 soils that showed the lowest binding strength for Ni impressively increased ${I}_{R}$ values even at the lower Ni loads.

**Table 5:** The relative binding intensity ($${I}_{R})$$ of nickel in the studied soils and the corresponding soil-BR mixtures (*n* = 2)

Ni (mg kg^−1^)	S1	S2	S3	S4	S5	S6	BR-S1	BR-S2	BR-S3	BR-S4	BR-S5	BR-S6
1	0.91	0.54	0.74	0.61	0.94	0.91	0.97	0.95	0.96	0.92	0.97	0.97
5	0.79	0.43	0.57	0.36	0.80	0.89	0.93	0.89	0.91	0.90	0.92	0.92
10	0.80	0.38	0.47	0.32	0.68	0.79	0.90	0.86	0.90	0.83	0.89	0.89
20	0.73	0.34	0.42	0.23	0.63	0.66	0.84	0.81	0.83	0.79	0.83	0.85
30	0.67	0.26	0.42	0.20	0.44	0.51	0.88	0.82	0.81	0.82	0.85	0.86
50	0.70	0.25	0.37	0.20	0.41	0.50	0.84	0.81	0.82	0.82	0.83	0.83
70	0.59	0.22	0.36	0.20	0.47	0.55	0.84	0.80	0.81	0.79	0.79	0.81
90	0.55	0.21	0.38	0.24	0.45	0.54	0.76	0.80	0.76	0.74	0.79	0.74

### Mobility factor

Though ${I}_{R}$ and ${M}_{F}$ (mobility factor) values are related, the ${M}_{F}$ values show greater sensitivity compared to the ${I}_{R}$ values in capturing variations in the easily available fraction of the metal. The high mobility and bioavailability of h.

eavy metals in soils with high ${M}_{F}$ values were well documented by Tang et al. (2017) and Yang and Hodson (2019). In the studied soils and soil-BR mixtures, the ${M}_{F}$ values increased as the added Ni amount increased and were much lower (up to three orders of magnitude) for the soil-BR mixtures than for the respective soils (Table 6). For the lower Ni loads, ${M}_{F}$ values in soils were below the medium risk threshold (30%) but ${M}_{F}$ values for increased Ni loads indicate high or very high risk. ${M}_{F}$ values for S2 and S4 acid soils showed medium to high mobility of Ni even at the lower metal loads. The definitive effect of BR on Ni mobility is well portrayed by the ${M}_{F}$ values calculated for soil-BR mixtures that regardless the initial Ni load were always < 10% pointing to intense Ni immobilization and mitigation of the potential hazards posed by Ni effluents to the environment and human health.

**Table 6:** Mobility factor $({M}_{F})$ of nickel in the studied soils and the corresponding soil-BR mixtures

Ni (mg kg^−1^)	S1	S2	S3	S4	S5	S6	BR-S1	BR-S2	BR-S3	BR-S4	BR-S5	BR-S6
1	7.09	15.75	2.59	16.80	3.03	8.24	0.68	0.73	0.46	1.89	0.62	0.62
5	18.11	21.26	7.53	43.03	14.25	9.43	0.87	1.66	0.86	1.49	1.21	0.80
10	16.91	26.28	10.94	49.56	24.18	20.49	0.82	2.49	1.21	2.48	1.17	1.25
20	22.14	38.98	14.73	66.31	24.70	34.06	1.00	3.21	2.32	2.51	1.54	1.52
30	30.37	55.34	24.88	71.90	49.07	49.46	0.88	3.64	2.85	2.70	1.49	1.44
50	27.53	50.14	36.73	73.71	53.05	50.74	1.08	3.29	2.54	2.31	1.56	1.81
70	40.92	57.86	36.59	72.59	45.75	46.16	1.37	3.47	3.97	2.76	1.95	2.50
90	43.94	63.60	35.79	65.63	44.74	47.74	1.43	4.04	6.36	3.82	1.60	8.06

## Conclusions

The study demonstrated that the addition of nickel (Ni) to bauxite residue (BR) did not alter its crystalline mineral phases, as confirmed by X-ray diffraction (XRD). However, Fourier-transform infrared (FTIR) spectroscopy revealed modifications in BR’s spectrum following Ni adsorption, indicating interaction of Ni with specific functional groups. Proposed mechanisms for Ni immobilization include the formation of Ni–Al layered double hydroxides (LDHs), surface precipitation of Ni(OH)₂, electrostatic adsorption on negatively charged sites, and diffusion into BR’s microporous matrix. Incorporation of BR into six different soils substantially increased Ni sorption capacity across all samples, resulting in nearly uniform adsorption curves. The distribution coefficient (*K*_*d*_) values for soil–BR mixtures increased by approximately four orders of magnitude, indicating a marked improvement in sorption efficiency. While the variability of soil properties played a significant role in Ni sorption in the absence of BR, the addition of BR masked the influence of individual soil characteristics, leading to a consistent increase in Ni retention and immobilization across all soils as over 80% of retained Ni extracted mainly from the residual and the oxidizable fractions. Furthermore, sequential extraction results revealed distinct shifts in Ni speciation. In untreated soils, increasing Ni concentrations elevated the exchangeable fraction, reaching up to 65% of total Ni. In contrast, BR addition significantly reduced the exchangeable and acid-soluble fractions while increasing the oxidizable and residual fractions. These changes reflect a notable reduction in Ni mobility and bioavailability. BR application also decreased the mobility factor (*M*_*F*_) of Ni and increased its relative binding intensity (IR), effectively transforming Ni from labile to non-labile forms. These findings support the application of BR as a stabilizing agent in Ni-contaminated soils and emphasize its potential to act as a long-term barrier against metal leaching, particularly under conditions involving small-scale site-specific application. Future research should employ X-ray absorption spectroscopy (XAS) to further elucidate the mechanisms of Ni immobilization in BR–soil systems. Evaluating a range of BR-to-soil ratios in soils with varying pH, amorphous Fe–Mn content, and clay composition will facilitate optimization of BR application. This study confirms that BR effectively reduces Ni mobility and toxicity, primarily through pH regulation and structural immobilization by minerals such as gibbsite and cancrinite. By transforming Ni into more stable, non-labile forms, BR functions as an efficient barrier against Ni contamination in soil environments.

## Supplementary Information

Below is the link to the electronic supplementary material.

ESM 1 DOCX (144KB)

## Data Availability

Data is available upon request.
