# Correction to: Nickel dynamics and immobilization in soil‑bauxite residue systems: insights from sequential extraction and FTIR analysis

**DOI:** 10.1007/s11356-025-36877-4

**Published:** 2025-08-16

**Authors:** Ioannis Zafeiriou, Dafni Ioannou, Panagiotis Angelopoulos, Evgenia Georgiou, Dionisios Gasparatos, Ioannis Massas

**Affiliations:** 1 Laboratory of Soil Science and Agricultural Chemistry, Department of Natural Resources Management & Agricultural Engineering, School of Environment & Agricultural Engineering, Agricultural University of Athens, 11855 Athens, Greece; 2 Laboratory of Metallurgy, School of Mining and Metallurgical Engineering, National Technical University of Athens, Zografou Campus, 9 Iroon Polytechniou Str., 15780 Athens, Zografou Greece

**Correction to: Environmental Science and Pollution Research (2025) 32:17730–17746**

10.1007/s11356-025-36701-z

The correct family name of the 3rd Author is "Angelopoulos" not "Aggelopoulos".

The Original article has been corrected.

