# Supplementary material for: Nickel dynamics and immobilization in soil-bauxite residue systems: insights from sequential extraction and FTIR analysis
Source: Environ Sci Pollut Res Int. 2025 Jul 5;32(29):17730–46. doi: 10.1007/s11356-025-36701-z (PMC12325433; doi:10.1007/s11356-025-36701-z)
Supplement: Supplementary file 1 — DOCX (144KB) [file 11356_2025_36701_MOESM1_ESM.docx]

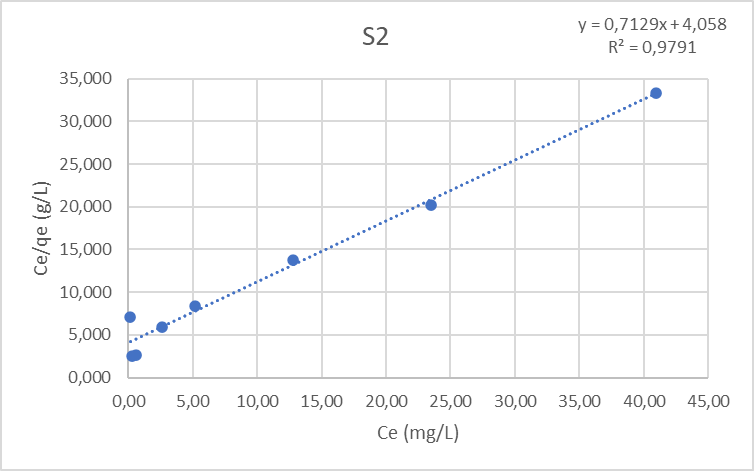

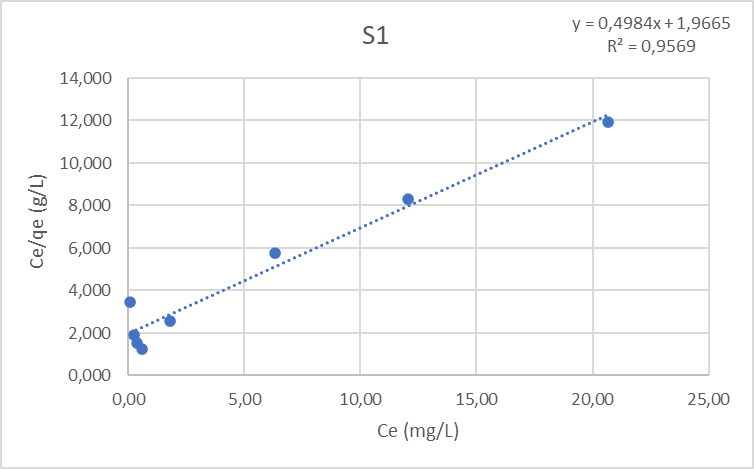


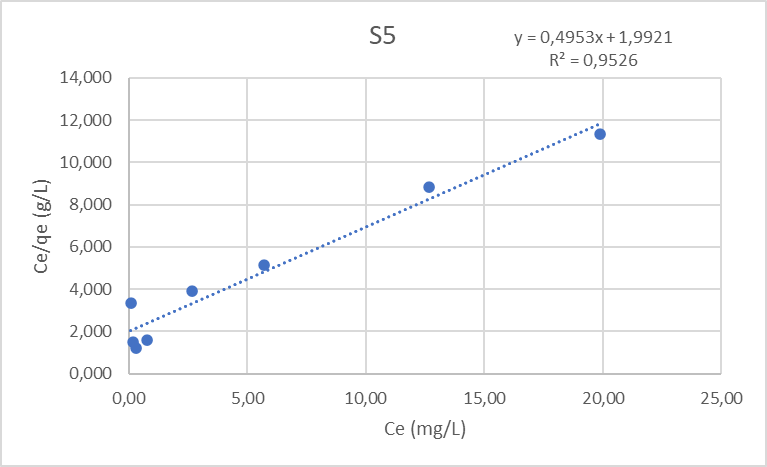

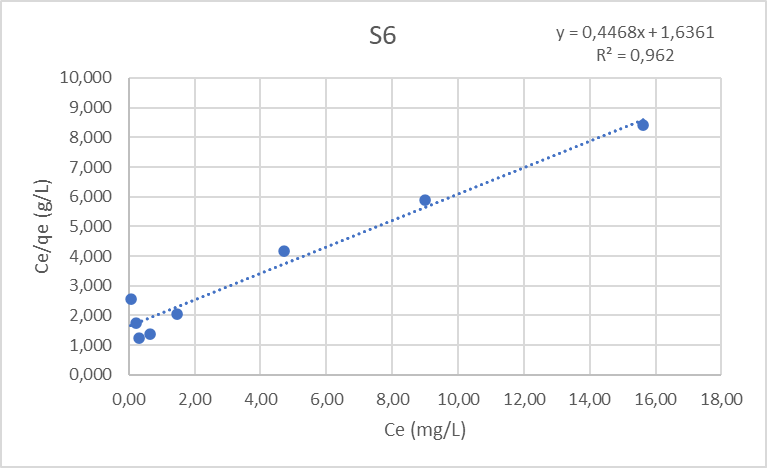


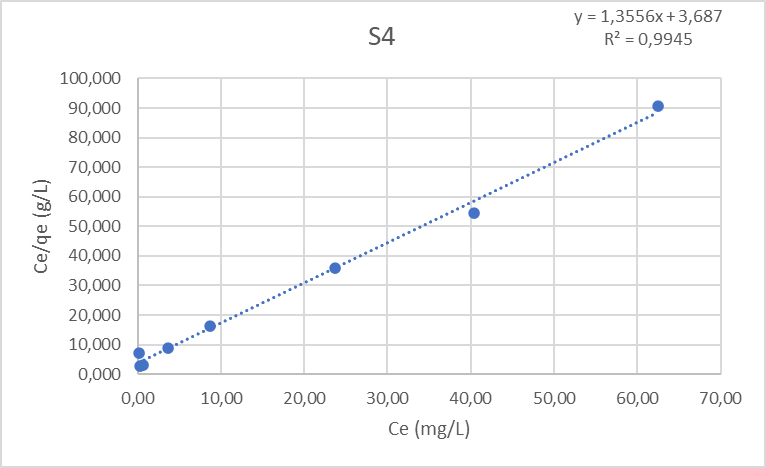

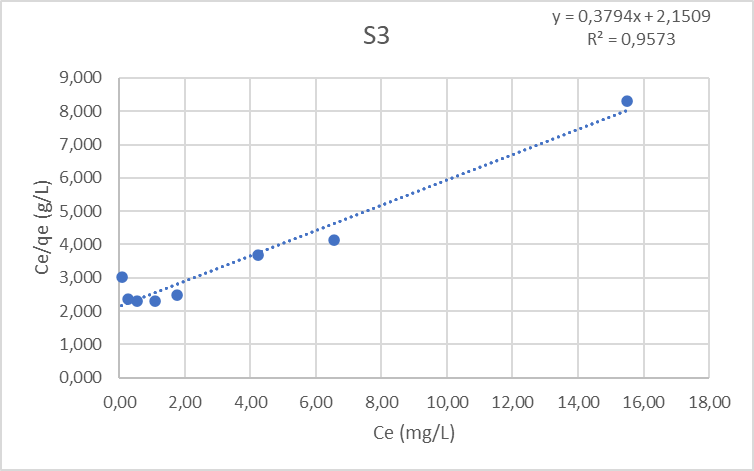


**Figure S1** Linear fitting plots of Langmuir model for the studied soils.


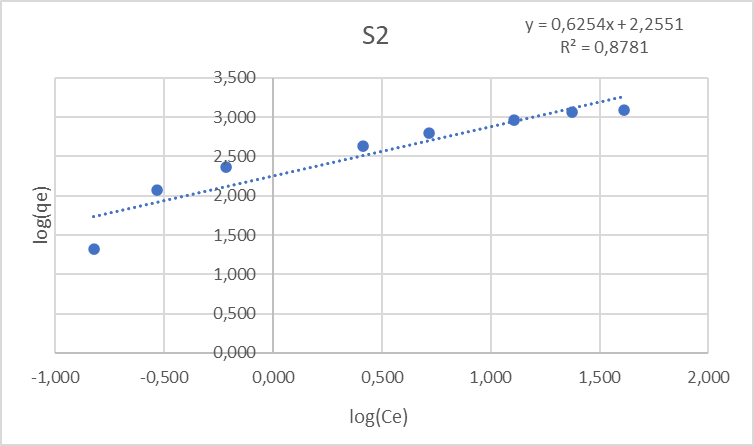

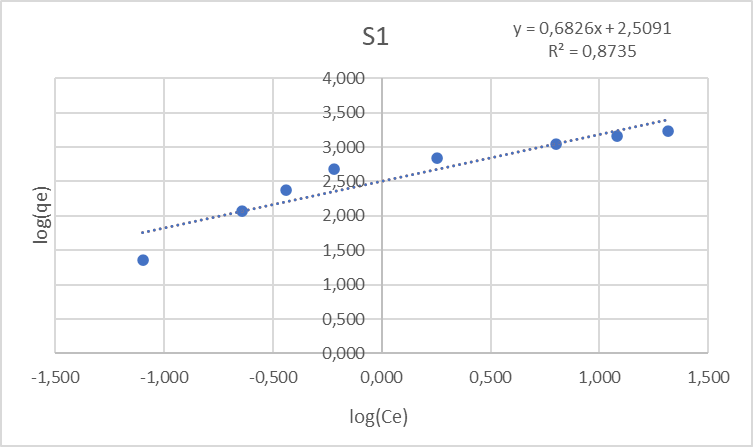


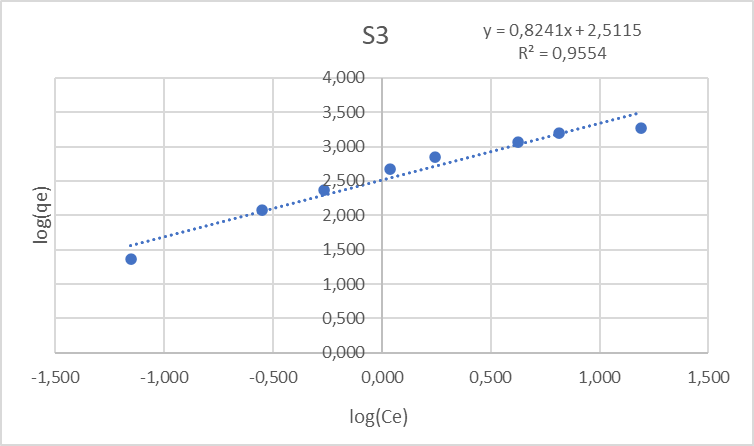

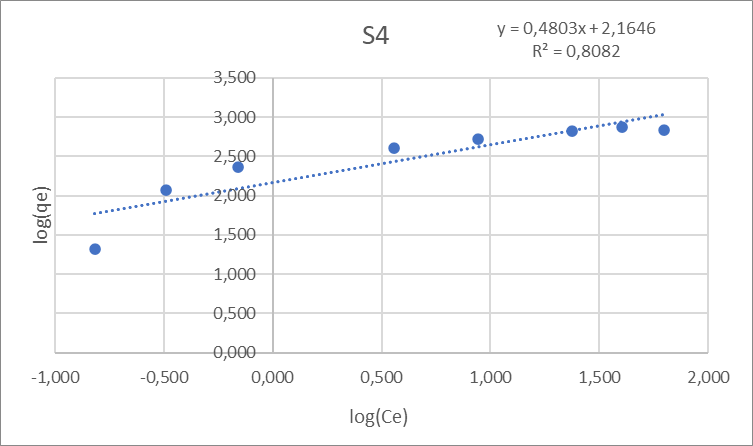


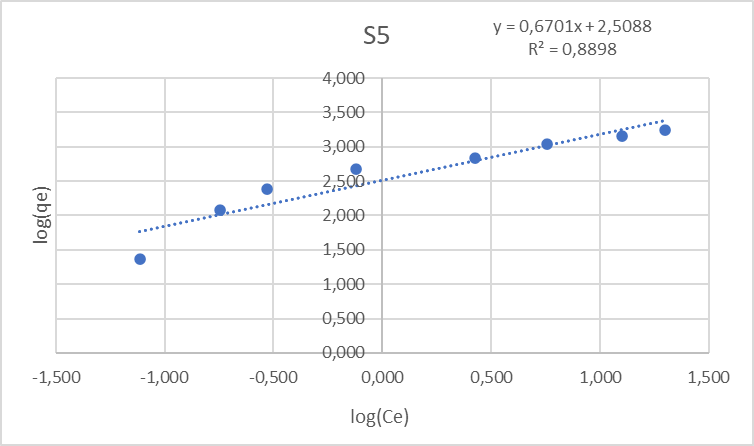


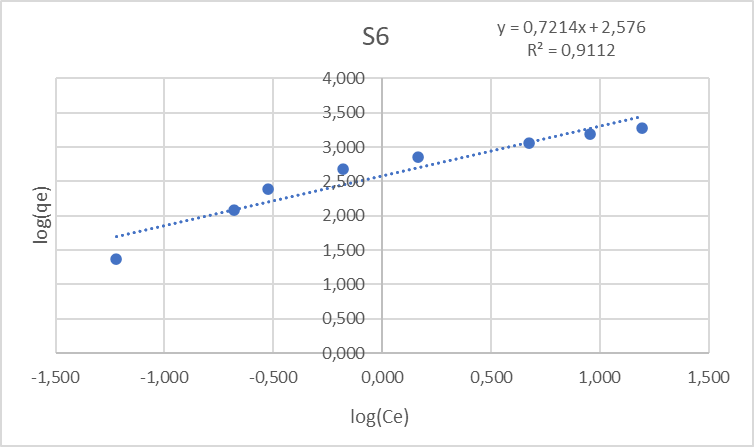


**Figure S2** Linear fitting plots of Freundlich model for the studied soils.

**Table S1** Initial (Co) and final (Ce) Ni concentration for the studied soils.

| **Co (mg L^-1^)** | **Ce (mg L^-1^)** | | | | | |
| --- | --- | --- | --- | --- | --- | --- |
|  | **S1** | **S2** | **S3** | **S4** | **S5** | **S6** |
| 1 | 0,08 | 0,15 | 0,07 | 0,15 | 0,08 | 0,06 |
| 5 | 0,23 | 0,29 | 0,28 | 0,32 | 0,18 | 0,21 |
| 10 | 0,37 | 0,61 | 0,54 | 0,69 | 0,30 | 0,30 |
| 20 | 0,60 | 2,60 | 1,09 | 3,62 | 0,76 | 0,66 |
| 30 | 1,80 | 5,21 | 1,75 | 8,73 | 2,66 | 1,46 |
| 50 | 6,30 | 12,78 | 4,23 | 23,68 | 5,72 | 4,72 |
| 70 | 12,06 | 23,52 | 6,54 | 40,34 | 12,66 | 9,00 |
| 90 | 20,68 | 40,90 | 15,48 | 62,46 | 19,90 | 15,62 |

**Table S2** Initial (Co) and final (Ce) Ni concentration for the studied soil-BR mixtures.

| **Co (mg L^-1^)** | **Ce (mg L^-1^)** | | | | | |
| --- | --- | --- | --- | --- | --- | --- |
|  | **S1-BR** | **S2-BR** | **S3-BR** | **S4-BR** | **S5-BR** | **S6-BR** |
| 1 | 0,12 | 0,14 | 0,13 | 0,10 | 0,11 | 0,11 |
| 5 | 0,18 | 0,34 | 0,42 | 0,18 | 0,22 | 0,50 |
| 10 | 0,21 | 0,29 | 0,28 | 0,26 | 0,20 | 0,22 |
| 20 | 0,25 | 0,36 | 0,26 | 0,24 | 0,15 | 0,18 |
| 30 | 0,11 | 0,20 | 0,16 | 0,17 | 0,07 | 0,08 |
| 50 | 0,39 | 0,59 | 0,23 | 0,54 | 0,33 | 0,27 |
| 70 | 1,74 | 1,82 | 0,89 | 2,31 | 1,53 | 1,37 |
| 90 | 3,70 | 5,66 | 2,17 | 6,21 | 4,29 | 2,27 |
